# Ethanol extract of *Vanilla planifolia* stems reduces PAK6 expression and induces cell death in glioblastoma cells

**DOI:** 10.1111/jcmm.70065

**Published:** 2024-09-04

**Authors:** Hui Hua Chang, Alice Y. W. Chang, Bing‐Chen Tsai, Yu‐Ju Chen, Sung‐Ghun Wu, Li‐Jyun Chen, Yi‐Xuan Lin, Yuan‐Shuo Hsueh

**Affiliations:** ^1^ Institute of Clinical Pharmacy and Pharmaceutical Sciences, College of Medicine, National Cheng Kung University Tainan Taiwan; ^2^ School of Pharmacy, College of Medicine National Cheng Kung University Tainan Taiwan; ^3^ Department of Pharmacy National Cheng Kung University Hospital, College of Medicine, National Cheng Kung University Tainan Taiwan; ^4^ Department of Pharmacy National Cheng Kung University Hospital, Dou‐Liou Branch Yunlin Taiwan; ^5^ Department of Physiology, College of Medicine National Cheng Kung University Tainan Taiwan; ^6^ Department of Medical Science Industries, College of Health Sciences Chang Jung Christian University Tainan Taiwan; ^7^ Graduate Institute of Medicine, College of Medicine, Kaohsiung Medical University Kaohsiung Taiwan; ^8^ Department of Physiology, School of Post Baccalaureate Medicine, College of Medicine Kaohsiung Medical University Kaohsiung Taiwan; ^9^ Center for Cancer Research Kaohsiung Medical University Kaohsiung Taiwan; ^10^ Department of Medical Research Kaohsiung Medical University Hospital Kaohsiung Taiwan

**Keywords:** differentially expressed genes, glioblastoma multiforme, PAK6, therapeutic targets, *Vanilla planifolia*

## Abstract

Glioblastoma multiforme (GBM) is a malignant tumour with a poor prognosis. Therefore, potential treatment strategies and novel therapeutic targets have gained increased attention. Our data showed that the ethanol extract of *Vanilla planifolia* stem (VAS) significantly decreased the viability and the colony formation of GBM cells. Moreover, VAS induced the cleavage of MAP1LC3, a marker of autophagy. Further RNA‐seq and bioinformatic analysis revealed 4248 differentially expressed genes (DEGs) between VAS‐treated GBM cells and the control cells. Protein–protein interactions between DEGs with fold changes less than −3 and more than 5 were further analysed, and we found that 16 and 9 hub DEGs, respectively, were correlated with other DEGs. Further qPCR experiments confirmed that 14 hub DEGs was significantly downregulated and 9 hub DEGs was significantly upregulated. In addition, another significantly downregulated DEG, p21‐activated kinase 6 (PAK6), was correlated with the overall survival of GBM patients. Further validation experiments confirmed that VAS significantly reduced the mRNA and protein expression of PAK6, which led to the abolition of cell viability and colony formation. These findings demonstrated that VAS reduced cell viability, suppressed colony formation and induced autophagy and revealed PAK6 and other DEGs as potential therapeutic targets for GBM treatment.

## 1 INTRODUCTION

Glioblastoma multiforme (GBM) is the most common malignant brain tumour in adults.^1^, ^2^ According to the World Health Organization (WHO) classification, GBM is defined as Grade IV astrocytoma, indicating that has the highest degree of malignancy and aggressiveness among astrocytoma.^3^ Although the incidence rate of GBM is relatively low, the disease as attracted researchers' attention due to its poor overall survival and high mortality rates after conventional surgery, chemotherapy and radiotherapy.^4^, ^5^ According to The Cancer Genome Atlas (TCGA) database, GBM is characterized by aberrant amplification or mutation of EGFR, PDGFRA, CDK4, NF1, HER2, TP53, PIK3R1 and TERT.^6^, ^7^ These findings prompted scientists to evaluate several inhibitors of EGFR or PDGFRA in vitro and in vivo for GBM treatment. Unfortunately, these clinical trials of EGFR inhibitors or PDGFRA inhibitors in GBM patients failed.^8^, ^9^, ^10^, ^11^, ^12^ Several potential targets, such as HSP90 and HDAC, and their specific inhibitors have been proven to have antitumor effects on GBM cells.^13^, ^14^ However, there is still an urgent need for new treatment options for GBM.

*Vanilla planifolia*, a type of vanilla orchid, is a major source of vanilla flavouring due to its high vanillin content.^15^ Vanillin is widely used as a crucial flavour and aromatic component in global cuisine. Numerous studies have explored its various functions, including its potential as an antitumor agent. Ho et al. and Ramadoss et al. showed that vanillin can induce apoptosis and arrest the cell cycle in colon cancer.^16^, ^17^ Other studies revealed that vanillin interacts with calcium/calmodulin‐dependent protein kinase IV and microtubule affinity‐regulated kinase 4, leading to mitochondrial damage, reactive oxygen species production and apoptosis.^18^, ^19^ Vanillin can modulate the PI3K, NFKB and STAT3/HIF1A signalling pathways and inhibit cell invasion and metastasis.^20^, ^21^, ^22^, ^23^ Furthermore, vanillin has been found to synergistically enhance the anticancer effects of cisplatin and doxorubicin.^24^, ^25^ Despite the recognized anticancer potential of vanillin, the detailed molecular mechanism underlying its tumour‐suppressive effects has yet to be fully elucidated.

The p21‐activated kinase (PAK) family comprises six serine/threonine kinase family members categorized into two groups (Group I [PAK1, PAK2 and PAK3] and Group II [PAK4, PAK5, PAK6]).^26^ PAKs have been demonstrated to mediate multiple cellular functions, including cell growth, cytoskeletal motility, cell proliferation, survival, and apoptosis.^27^, ^28^ PAKs were originally identified as proteins that are activated by the Rho GTPases Rac1 and Cdc42. However, the overexpression of PAKs is often observed in some cancer types.^29^ Therefore, PAKs are potential diagnostic biomarkers and therapeutic targets for cancer treatment. Several studies have shown that Group II PAK members, including PAK6, play essential roles in oncogenic signalling pathways. PAK6 regulates several cell functions, such as apoptosis, the cell cycle, cell growth, cell migration and invasion. High PAK6 mRNA expression has been observed in lung cancer, colon cancer, ovarian cancer and prostate cancer.^30^ PAK6 expression in prostate cancer is even increased when androgen deprivation therapy fails.^31^ The combination of a PAK6 inhibitor and docetaxel significantly reduced the viability of prostate cancer cells.^32^ Therefore, PAK6 is also a potential therapeutic target for cancer treatment.

In this study, we aimed to evaluate the antitumor effect of *Vanilla planifolia* on GBM cells. The leaves, stems and pods of *Vanilla planifolia* were extracted with ethanol or water. Three GBM cell lines, patient‐derived temozolomide (TMZ)‐resistant GBM P#5 TMZ‐R cells, T98G cells and U‐87 MG cells, were used to evaluate the antitumor activity of the ethanol extract of *Vanilla planifolia* stem (VAS). Then, RNA‐seq was performed to explore the differentially expressed genes (DEGs) between control and VAS‐treated cells. Further qPCR was used to validate the RNA‐seq findings. Our data showed that VAS reduced the viability and suppressed the colony formation of GBM cells. Moreover, VAS induced MAP1LC3 cleavage and altered the expression of genes involved in the cell cycle, apoptosis and autophagy. Further experiments revealed that these DEGs and PAK6 may play important roles in VAS‐induced cell death and are potential therapeutic targets for GBM treatment.

## 2 MATERIALS AND METHODS

### 2.1 Extraction of *Vanilla planifolia*

*Vanilla planifolia* was planted and provided by Shing‐Kuan Wu (Chang Jung Christian University). The leaves, stems and pods of *Vanilla planifolia* were harvested and extracted with ethanol or water. The extracts were filtered and evaporated using a rotary evaporator. The crude extracts were freeze‐dried to ensure that the sample was free of water. The crude extracts of ethanol and water were resolved in DMSO and ddH_2_O, respectively.

### 2.2 Cell culture, chemicals and antibodies

The U‐87 MG and T98G GBM cell lines were purchased from BCRC (#60360, Taiwan) and JCRB (JCRB9041, Japan), respectively. P#5 TMZ‐R, a patient‐derived GBM cell line with temozolomide resistance, was kindly provided by Dr. Jian Ying Chuang (Taipei Medical University, Taiwan). In accordance with the methods of a previous study, cells were cultured in Dulbecco's modified Eagle's medium (DMEM) supplemented with 10% fetal bovine serum (FBS; HyClone, USA), 100 U/mL penicillin and 0.1 mg/mL streptomycin (GeneDirex, Taiwan) and maintained in a humidified incubator at 37°C with 5% CO_2_.^13^ Specific antibodies against MAP1LC3 (Cell Signaling Technology, Danvers, MA) and GAPDH (Santa Cruz Biotechnology, Dallas, TX) were used.

### 2.3 Cell viability assay

This assay was performed following previous methods.^33^ Cells in 24‐well plates were exposed to ethanol or water extracts of *Vanilla planifolia* at the specified doses. After 5 days, the cells were stained with methylene blue dye. The dye was dissolved, and the absorbance was measured at a wavelength of 595 nm by a SpectraMax iD3 Microplate Reader (Molecular Devices). Cell viability was assessed by measuring the absorbance and normalising the results to that of the DMSO‐only control group. All the experimental data points were obtained from triplicate wells within each plate and were replicated in a minimum of three plates. The data are presented as the mean ± standard error of the mean (SEM).

### 2.4 Colony formation assay

This assay was performed following the protocol of a previous study.^33^ After the cells were seeded in 6‐well plates, they were treated with the extracts for 24 h. The medium was replaced with fresh growth medium without extracts, after which the cells were incubated for 2 weeks. Colonies were visualized by staining with 0.5% methylene blue, and the number of colonies was determined and normalized to that of the control group.

### 2.5 Immunoblotting

This assay was performed following previous methods.^33^ The cells were lysed with CelLyticTM M cell lysis buffer containing protease and phosphatase inhibitors. The protein concentration was determined using the Bio‐Rad® protein assay dye reagent concentrate (Cat. #500‐0006). Equal amounts of total protein were denatured, separated by sodium dodecyl sulfate–polyacrylamide gel electrophoresis (SDS–PAGE), and transferred to PVDF membranes. The specific proteins on the PVDF membrane were identified by primary antibodies and HRP‐conjugated secondary antibodies, visualized using enhanced chemiluminescence (ECL) reagents (PerkinElmer) and imaged using an iBright imaging system (Thermo Fisher Scientific®). The band density was analysed using ImageJ software.

### 2.6 RNA extraction

The procedure followed the standard protocol of the Quick‐RNA™ Miniprep Kit (ZYMO RESEARCH®). Briefly, TRIzol Reagent was used to lyse VAS‐treated cells. Total RNA was then purified using Zymo‐Spin™ columns and quantified using NanoDrop™ spectrophotometers (Thermo Fisher Scientific®).

### 2.7 RNA sequencing and bioinformatic analysis

RNA sequencing was performed by Genomics, Inc. (Taiwan) using the Illumina NovaSeq platform following previous methods.^13^, ^14^ The quality of the RNA was assessed by calculating RNA integrity number (RIN) scores using an Agilent 2100 Bioanalyzer. The sequenced RNA was aligned to the human genome (hg19). Gene expression levels were quantified using RSEM software. Differentially expressed genes (DEGs) were identified using EBSeq with a threshold false discovery rate (FDR) of 0.05. Gene Ontology (GO) enrichment analysis and Kyoto Encyclopedia of Genes and Genomes (KEGG) pathway analysis were also conducted. Additionally, the protein–protein interaction (PPI) network of DEGs was visualized using the Search Tool for the Retrieval of Interacting Genes/Proteins (STRING) website (https://string‐db.org/cgi/input?sessionId=bBRBaHkRJB8j&input_page_show_search=on) to gain insight into the involvement of these genes.

### 2.8 Quantitative analysis of mRNA

One microgram of total RNA and the ReverTra Ace Set (PURIGO, Taiwan) were used to synthesize cDNA. For mRNA expression quantification, real‐time PCR (qPCR) was performed using THUNDERBIRD® SYBR® qPCR Mix (TOYOBO, Japan), and the results were analysed with a StepOnePlus™ Real‐Time PCR System (Thermo Fisher Scientific®). The primers used for qPCR can be found in Table S1. Each experiment was conducted in triplicate, and the data are presented as the mean ± standard error of the mean (SEM).

### 2.9 Lentivirus infection

Cells at approximately 70% confluence in 6‐well plates were infected with lentivirus containing pLKO.1‐shPAK6#1 (shPAK6#1), pLKO.1‐shPAK6#2 (shPAK6#2), or scramble control (Sinica, Taiwan) for 24 h. Infected cells incubated with growth media and then collected for subsequent experiments. The effects of PAK6 downregulation at the mRNA level were determined by qPCR and the effects at the protein level were determined by immunoblotting after the appropriate treatment time.

### 2.10 Statistical analyses

The data were analysed using the Statistical Package for the Social Sciences (SPSS, Inc.), version 16 for Windows. All the data are presented as the mean ± standard error of the mean (SEM). One‐way analysis of variance (ANOVA) was used to compare the means among different groups and then Bonferroni post hoc correction was performed. The threshold for statistical significance was set as *p < 0.05.

## 3 RESULTS

### 3.1 The ethanol extract of *Vanilla planifolia* stem reduces viability, suppresses colony formation and induces autophagy in GBM cells

**FIGURE 1:**
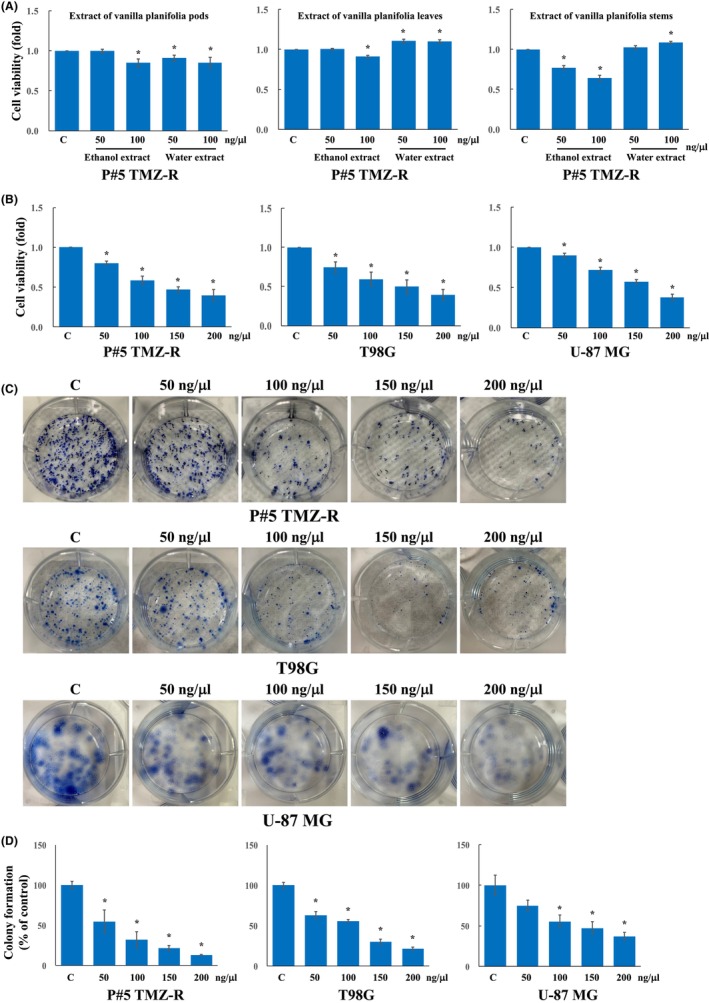
The antitumor effect of crude extracts of *Vanilla planifolia* on GBM cells. The cells were incubated with water or ethanol extracts of *Vanilla planifolia* pods, leaves, or stems at the indicated doses and then analysed via cell viability assays (A). The cells were treated with VAS at the indicated doses and then analysed via cell viability assays (B) and clonogenic assays (C). The IC_50_ values were determined by plotting the growth relative of the treated and control cells. The colonies were counted manually, and the results were compared to those of the vehicle control (D). All the experiments were repeated at least three times. The data are expressed as the means ± SEMs of three or more independent experiments. *p < 0.05.

**FIGURE 2:**
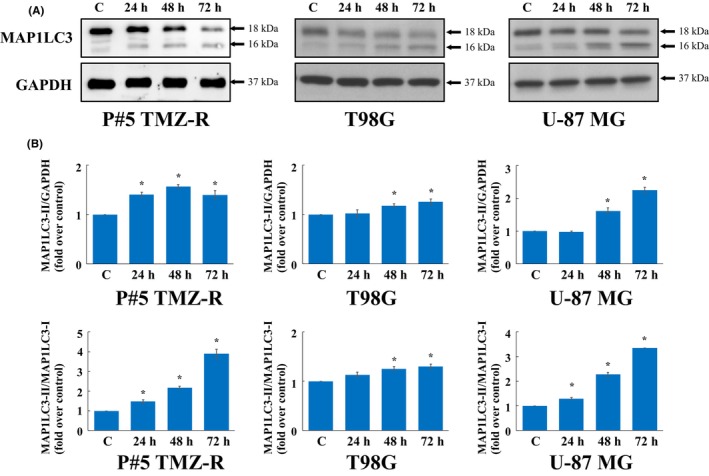
Effect of VAS on autophagy in GBM cells. The cells were incubated with 200 ng/μl VAS for the indicated times. The cells were analysed by immunoblotting with specific antibodies (A). GAPDH was used as an internal control. The quantification of each band is shown in B. All the experiments were repeated at least three times. The data are expressed as the means ± SEMs of three or more independent experiments. *p < 0.05.

In this study, three GBM cell lines, namely, patient‐derived temozolomide (TMZ)‐resistant GBM P#5 TMZ‐R cells, T98G cells, and U‐87 MG cells, were used to evaluate the antitumor activities of the ethanol extract and water extract of *Vanilla planifolia*. First, P#5 TMZ‐R cells were treated with the pods, leaves, and stems of *Vanilla planifolia* crude extracts of ethanol and water at 50 and 100 ng/μl. Figure 1A shows that the ethanol and water extracts of *Vanilla planifolia* pods and leaves had only slight inhibitory effects. Interestingly, the ethanol extract of *Vanilla planifolia* stem (VAS) significantly reduced the cell viability of P#5 TMZ‐R at 50 and 100 ng/μl, but the water extract of *Vanilla planifolia* stem did not inhibit cell viability. Then, to determine the IC_50_, the cells were further treated with VAS at 50, 100, 150 and 200 ng/μl. Figure 1B shows that VAS effectively decreased the viability of P#5 TMZ‐R cells (IC_50_: 137 ng/μl), T98G cells (IC_50_: 150 ng/μl) and U‐87 MG cells (IC_50_: 168 ng/μl). Furthermore, 200 ng/μl VAS significantly inhibited the colony formation of P#5 TMZ‐R, T98G and U‐87 MG cells (Figure 1C,D). Further analysis of the cell death pathway after treatment with 200 ng/μl VAS for 72 h revealed that MAP1LC3‐II conversion occurred more readily in the treatment groups than in the control groups of P#5 TMZ‐R, T98G and U‐87 MG cells (Figure 2A,B). Taken together, these data indicate that VAS treatment induces autophagy and reduces the viability and colony formation of GBM cells.

### 3.2 Between VAS‐treated GBM cells and control GBM cells, there were 4248 DEGs, 14 significantly downregulated DEGs and 9 significantly upregulated DEGs according to qPCR

**FIGURE 3:**
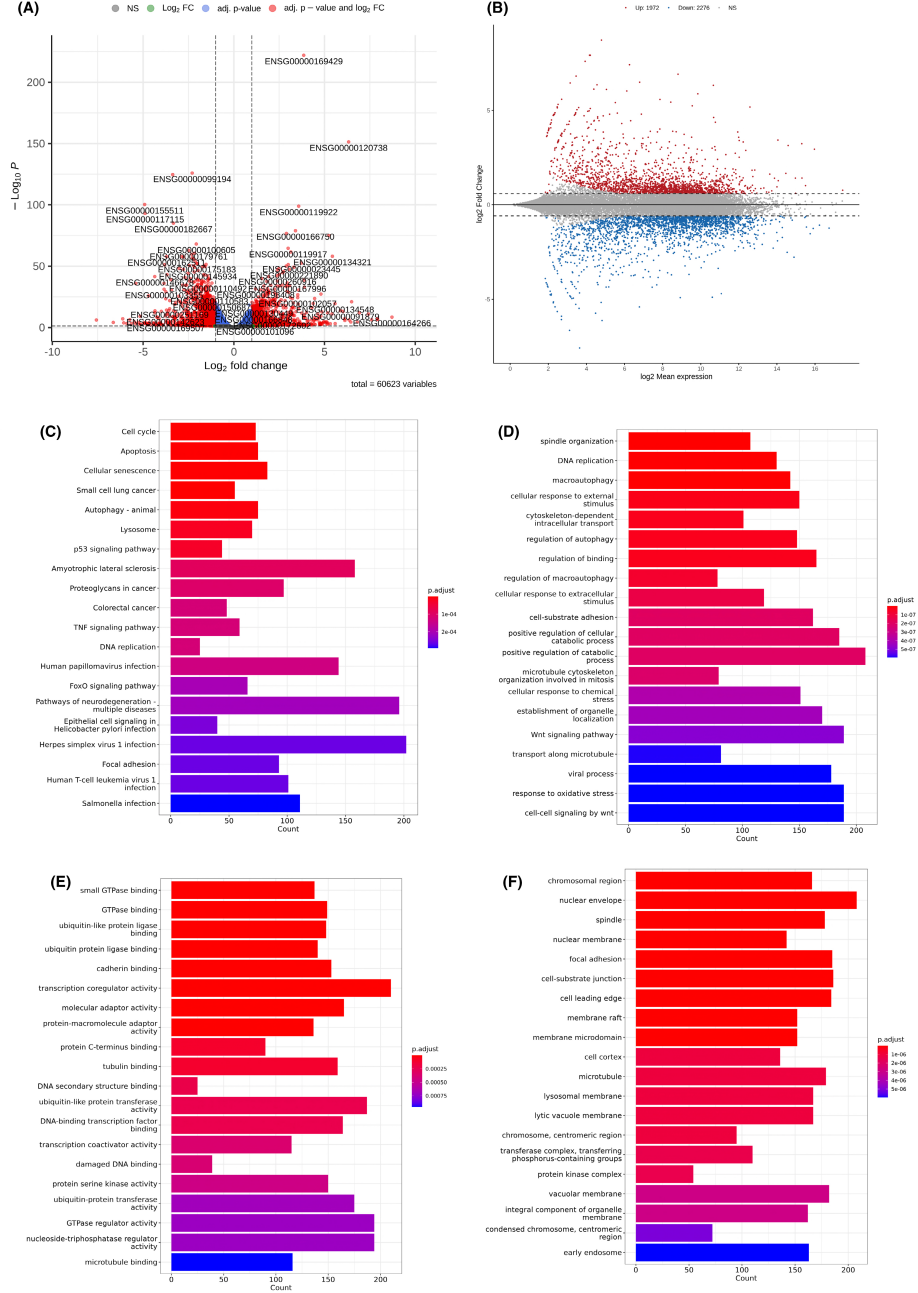
GO and KEGG analyses of the DEGs identified by RNA‐seq after VAS treatment. Volcano plot (A) and MA plot (B) of DEGs in P#5 TMZ‐R cells treated with 200 ng/μl VAS for 72 h. DEGs with *p* values less than 0.05 were further analysed by KEGG and GO analyses. (C) The results of the analysis of the top 20 pathways associated with the DEGs. (D) The top 20 biological process (BP) terms in the enrichment analysis. (E) The top 20 molecular function (MF) terms in the enrichment analysis. (F) The top 20 cellular component (CC) terms in the enrichment analysis.

Next, to verify which VAS‐related genes led to cell death, RNA‐seq was used to measure mRNA expression after VAS treatment. Based on our findings, the cells were treated with 200 ng/μl VAS and analysed via RNA‐seq. The transcript levels were compared between the 200 ng/μl VAS treatment group and the vehicle control group. The volcano plot and MA plot of the DEGs between the two groups are shown in Figure 3A,B. Between the VAS group and the vehicle control group, 1972 DEGs were upregulated (*p* < 0.05, log2‐fold change >1), and 2276 DEGs were downregulated (*p* < 0.05, log2‐fold change < −1). Then, we used KEGG enrichment and GO enrichment analyses to determine the functional annotations of these genes to investigate the involvement of the genes that were differentially expressed after VAS treatment; the annotations were grouped into biological process (BP), molecular function (MF), and cellular component (CC) categories. KEGG analysis revealed that the cell cycle, apoptosis, cellular senescence, autophagy and lysosome were the five most enriched terms (Figure 3C). GO analysis of the BP enrichment analysis revealed that spindle organization, DNA replication and macroautophagy were the three most enriched terms (Figure 3D). In the MF category of the GO analysis, small GTPase binding, GTPase binding, ubiquitin‐like or ubiquitin protein ligase binding and cadherin were the top enriched terms (Figure 3E). The terms associated with CC may be linked to the chromosomal region, nuclear envelope and spindle (Figure 3F). Taken together, these enrichment analysis results implied that VAS affects critical functions and pathways related to survival and proliferation in GBM cells.

**FIGURE 4:**
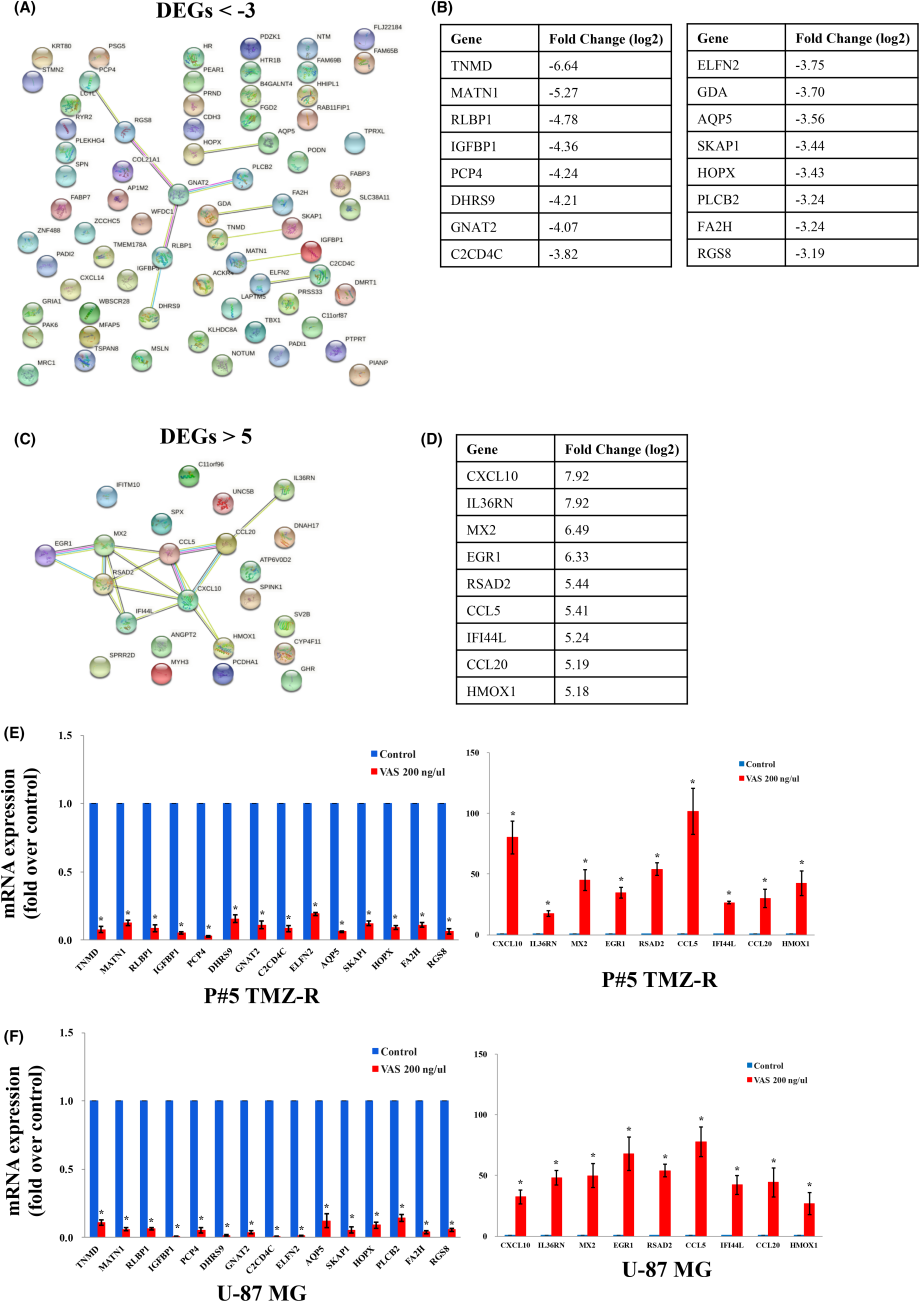
The mRNA expression of DEGs selected according to the PPI network in GBM after VAS treatment. (A) Eighty‐nine DEGs were selected by the criteria of log2PostFC <−3 and *p* < 0.05, and 16 DEGs were further selected by screening their correlations with each other in the STRING database. (C) Twenty‐three DEGs were selected by the criteria of log2PostFC >5 and *p* < 0.05, and 9 DEGs were selected by screening their correlations with each other in the STRING database. The fold change (log2) from RNA‐seq of these hub DEGs is shown in B and D. Cells were treated with VAS at 200 ng/μl for 72 h, and then the mRNAs were harvested. The mRNA expression levels of the downregulated and upregulated hub DEGs mentioned above were confirmed in P#5 TMZ‐R cells (E) and U‐87 MG cells (F) by qPCR. GAPDH was used as an internal control for qPCR. The data are expressed as the means ± SEMs of three or more independent experiments. *p < 0.05.

Tables 1 and 2 showed the top 10 downregulated and upregulated DEGs, respectively. To further explore the most important DEGs related to GBM patient survival after VAS treatment, we constructed a protein–protein interaction (PPI) network. Eighty‐nine downregulated DEGs with log2‐fold changes < −3 were identified via the STRING database, and 16 hub DEGs were correlated with one another: *TNMD*, *MATN1*, *RLBP1*, *IGFBP1*, *PCP4*, *DHRS9*, *GNAT2*, *C2CD4C*, *ELFN2*, *GDA*, *AQP5*, *SKAP1*, *HOPX*, *PLCB2*, *FA2H* and *RGS8* (Figure 4A). The fold changes in the expression of these hub DEGs, as determined by RNA‐seq, are shown in Figure 4B. Similarly, nine upregulated DEGs among 23 DEGs with log2‐fold change >5 were correlated with one another according to STRING analysis; these included *CXCL10*, *IL36RN*, *MX2*, *EGR1*, *RSAD2*, *CCL5*, *IFI44L*, *CCL20* and *HMOX1* (Figure 4C). The fold changes in the upregulated DEGs, as determined by RNA‐seq, are shown in Figure 4D.

**TABLE 1:** Top 10 downregulated DEGs.

Gene	Fold change (log2)
AQP5‐AS1	−7.57
TNMD	−6.64
TRDC	−6.12
LOC100506271	−6.07
C11orf87	−5.89
PRND	−5.74
G3BP1P1	−5.65
PAK6	−5.41
LINC01836	−5.40
MATN1	−5.27

**TABLE 2:** Top 10 upregulated DEGs.

Gene	Fold change (log2)
SPINK1	8.71
CXCL10	7.91
IL36RN	7.91
LOC729654	7.59
CYP4F11	7.41
UNC5B	6.91
DNAH17	6.81
MX2	6.49
SPRR2D	6.35
EGR1	6.32

To further validate the expression of these hub DEGs, a qPCR experiment was performed in P#5 TMZ‐R cells and U‐87 MG cells. Figure 4E shows that the mRNA expression levels of *TNMD*, *MATN1*, *RLBP1*, *IGFBP1*, *PCP4*, *DHRS9*, *GNAT2*, *C2CD4C*, *ELFN2*, *AQP5*, *SKAP1*, *HOPX*, *FA2H*, and *RGS8* were significantly reduced after VAS treatment in both P#5 TMZ‐R cells and U‐87 MG cells. Moreover, VAS also significantly induced the mRNA expression of *CXCL10*, *IL36RN*, *MX2*, *EGR1*, *RSAD2*, *CCL5*, *IFI44L*, *CCL20*, and *HMOX1* in both P#5 TMZ‐R cells and U‐87 MG cells. (Figure 4F). Taken together, these findings indicate that VAS could influence the expression of numerous genes and mediate cell death in GBM cells.

### 3.3 PAK6 plays a role in VAS‐induced cell death in GBM cells

**FIGURE 5:**
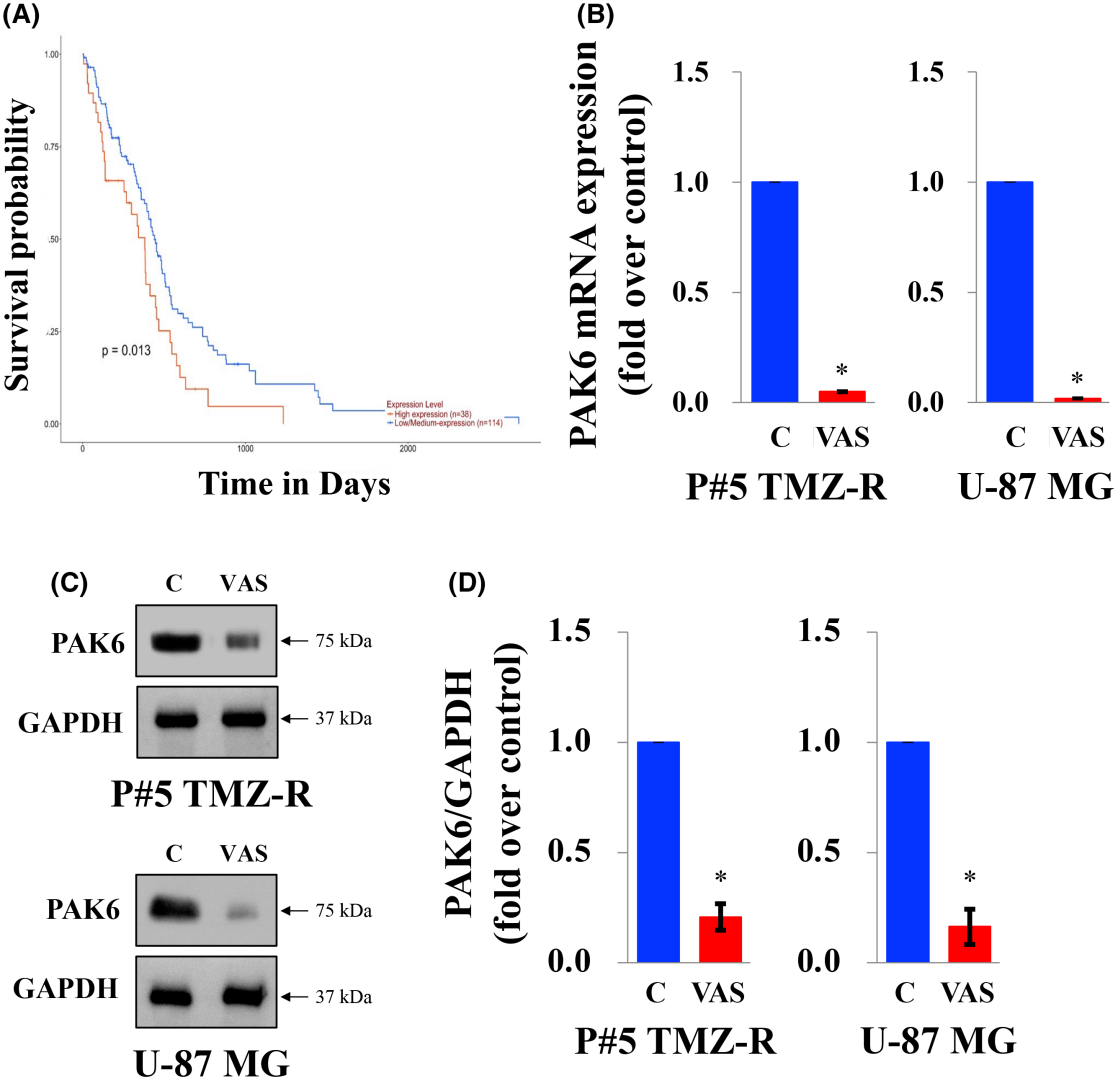
The role of PAK6 in VAS‐treated GBM cells. (A) Overall survival probabilities of GBM patients with high expression and low/medium expression of PAK6 according to analysis of the TCGA database. GBM cells were treated with VAS at 200 ng/μl for 72 h and analysed by qPCR (B) and immunoblotting for PAK6 (C). The quantification of each band in C is shown in D. ACTIN and GAPDH were used as internal controls for immunoblotting and qPCR, respectively. The data are expressed as the means ± SEMs of three or more independent experiments. *p < 0.05.

**FIGURE 6:**
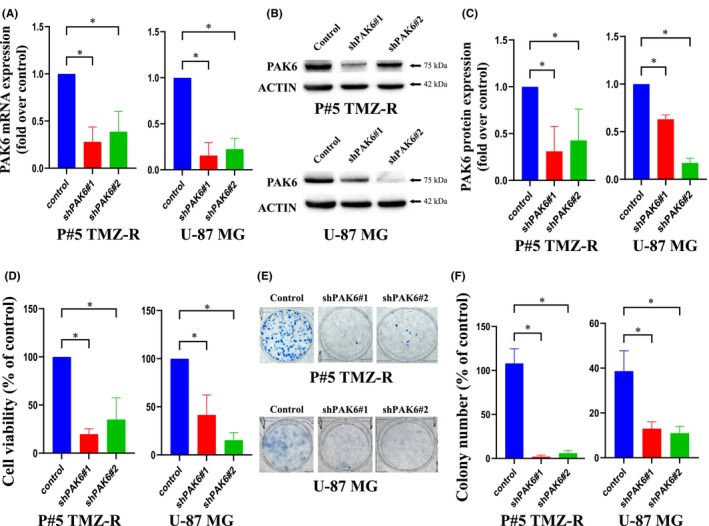
The role of PAK6 in the survival of GBM cells. Cells were infected with shPAK6#1 or shPAK6#2, and the mRNA (A) and protein (B) expression levels were determined via qPCR and immunoblotting, respectively. The quantification of each band in B is shown in C. PAK6‐silenced cells or scramble cells were analysed for cell viability (D) or colony formation (E). The colonies were counted manually, and the results were compared to those of the vehicle control (F). ACTIN and GAPDH were used as internal controls for immunoblotting and qPCR, respectively. The data are expressed as the means ± SEMs of three or more independent experiments. *p < 0.05.

In addition to performing PPI analysis, we also assessed the correlation between the DEGs and clinical outcomes. Our findings revealed a significant reduction in PAK6 mRNA levels after VAS treatment (log2‐fold change = −5.4). Moreover, we observed that GBM patients with high expression of PAK6 had a poorer prognosis than those with low expression according to analysis of the TCGA database (Figure 5A). Consequently, PAK6 was identified as the most prominently downregulated DEG and was correlated with patient survival, suggesting that it is a potential therapeutic target in GBM cells. According to the literature, PAK6 belongs to the PAK family and mediates various cellular functions. Notably, PAK6 is overexpressed in certain cancer types, suggesting that PAK6 could serve as a potential therapeutic target for cancer treatment. To further validate the impact of VAS on PAK6, we found that the mRNA and protein expression levels of PAK6 were significantly reduced after VAS treatment at a concentration of 200 ng/μl in both P#5 TMZ‐R and U‐87 MG cells (Figure 5B–D). To explore the role and mechanism of PAK6 in VAS‐induced GBM cell death, we employed shRNA against PAK6 to downregulate its mRNA and protein expression. As a result, both the mRNA and protein levels of PAK6 were significantly reduced in GBM cells treated with shPAK6#1 or shPAK6#2 (Figure 6A–C). Subsequently, we conducted cell function assays in PAK6‐silenced P#5 TMZ‐R and U‐87 MG cells, which revealed that cell viability was decreased and colony formation was inhibited in these cells compared to parental cells (Figure 6D–F). Collectively, these findings suggest that PAK6 may play a role in VAS‐induced cell death.

## 4 DISCUSSION

In the present study, we found that neither the ethanol extract nor the water extract of *Vanilla planifolia* pods or leaves had obvious inhibitory effects on GBM cells. The water extract of *Vanilla planifolia* stems had little inhibitory effect on cell viability. Interestingly, VAS significantly reduced the viability and colony formation ability of GBM cells. VAS also induced MAP1LC3B cleavage, suggesting that VAS‐induced cell death might occur via autophagy activation. Further RNA‐seq analysis after VAS treatment revealed that the levels of 1972 DEGs were upregulated and those of 2276 DEGs were downregulated. KEGG and GO analyses revealed that these DEGs were involved in the cell cycle, apoptosis, autophagy, spindle organization, and DNA replication. Further analysis of PPI with fold changes in DEGs less than −3 and greater than 5 revealed 16 and 9 hub DEGs, respectively. Further qPCR experiments revealed that 14 hub DEGs were significantly downregulated and 9 hub DEGs were significantly upregulated.

The 14 downregulated hub DEGs after VAS treatment included *TNMD*, *MATN1*, *RLBP1*, *IGFBP1*, *PCP4*, *DHRS9*, *GNAT2*, *C2CD4C*, *ELFN2*, *AQP5*, *SKAP1*, *HOPX*, *FA2H* and *RGS8*. Several studies have shown the correlation of these hub genes with cancer. Rakha et al. showed that the expression level of retinaldehyde binding protein 1 (RLBP1) was associated with tumour grade in invasive breast cancer.^34^ High insulin‐like growth factor binding protein 1 (IGFBP1) expression is significantly associated with poorer survival and relapse‐free survival in patients with gastric cancer.^35^ Purkinje cell protein 4 (PCP4), also called PEP19, has been demonstrated to promote the migration, invasion and adhesion of human breast cancer MCF‐7 and T47D cells.^36^ Dehydrogenase/reductase 9 (DHRS9) overexpression is correlated with a poor response to concurrent chemoradiotherapy and a poor prognosis in rectal cancer patients.^37^ Extracellular leucine‐rich repeat and fibronectin type III domain containing 2 (ELFN2) is highly expressed in astrocytoma patients and significantly correlated with overall survival.^38^ The expression level of aquaporin 5 (AQP5) in breast cancer is associated with lymph node metastasis and a poor prognosis.^39^ According to a transcriptome‐wide association study, low src kinase‐associated phosphoprotein 1 (SKAP1) expression in blood increases endometrial cancer risk.^40^ Homeodomain‐only protein homeobox (HOPX) downregulation by epigenetic regulation leads to aggressive breast cancer.^41^ Dai et al. reported that fatty acid 2‐hydroxylase (FA2H) is downregulated in triple‐negative breast cancer both in vitro and in the clinic.^42^ These authors further demonstrated that FA2H suppresses cancer stemness in breast cancer cells by inhibiting the STAT3/IL6 axis and NFkB signalling. The mRNA and protein expression levels of regulator of G protein signalling 8 (RGS8) in normal tissues are greater than those in thyroid carcinoma tissues.^43^ Bai et al. also showed that high expression of RGS8 in patients with thyroid carcinoma is associated with better survival outcomes than low expression.

In addition to the downregulated hub DEGs, there were 9 hub DEGs that were upregulated by VAS; they included *CXCL10*, *IL36RN*, *MX2*, *EGR1*, *RSAD2*, *CCL5*, *IFI44L*, *CCL20* and *HMOX1*. The CXCL9, CXCL10 and CXCL11/CXCR3 axes have been demonstrated to regulate immune cell migration, differentiation, and activation, leading to tumour suppression.^44^ Interleukin 36 receptor antagonist (IL36RN) can inhibit the activity of interleukin‐36 by binding to its receptor IL1RL2 and mediating inflammation. The expression of the IL‐36 family member mRNA and protein is significantly increased in colorectal cancer tissue compared to adjacent non‐tumour tissues.^45^ MX2 was obviously downregulated in both GBM patients and GBM cell lines.^46^ Moreover, MX2 overexpression markedly reduced the proliferation, migration, and invasion of glioma cells. Early growth response‐1 (EGR1) downregulation in prostate cancer cells reduces both the number and size of metastases but does not affect tumour growth, suggesting that EGR1 regulates angiogenic factors and promotes metastasis.^47^ The Cancer Genome Atlas (TCGA) gene expression profile and Kaplan–Meier analysis revealed that high expression of radical s‐adenosyl methionine domain containing 2 (RSAD2) in patients with oral cancer was associated with a better prognosis.^48^ Melese et al. reported that targeting CCL5 or CCL5 receptors on immunosuppressive cells to alter the immune microenvironment promoted lung cancer progression and immunotherapy insensitivity.^49^ In the clinic, the expression level of interferon‐induced protein 44‐like (IFI44L) is significantly reduced in HCC tumour tissues.^50^ Low IFI44L expression was also correlated with increased tumour size, disease relapse, advanced disease stage and poor clinical survival in HCC patients. The CCL20‐CCR6 axis promotes cancer progression directly by enhancing the migration and proliferation of cancer cells and indirectly by remodelling the tumour microenvironment through immune cell control.^51^ HMOX1 is highly expressed in a variety of cancers, including lung, breast, colorectal and glioblastoma.^52^, ^53^

In addition to these 14 downregulated hub DEGs and 9 upregulated hub DEGs, PAK6 was another significantly downregulated DEG and was correlated with the overall survival of GBM patients. PAK6 has been proven to promote homologous recombination, enhancing chemoresistance to oxaliplatin through ATR/CHK1 signalling in gastric cancer.^54^ Huang et al. reported that FAT atypical cadherin 4 (FAT4) knockout in the normal human hepatic cell line L02 activated PAK6 and its downstream WNT/β‐catenin signalling to promote tumour growth.^55^ Pharmacological inhibition of PAK6 perturbs the RAS/MAPK pathway and mitochondrial activity, sensitising therapy‐resistant leukaemic stem cells in chronic myeloid leukaemia to tyrosine kinase inhibitors.^56^ The PAK6‐SIRT4‐ANT2 complex has been demonstrated to be involved in mitochondrial apoptosis in prostate cancer cells.^57^ Additionally, further investigation revealed that PAK6 could phosphorylate AR, leading to AR degradation and cell death in prostate cancer.^58^ Downregulation of PAK6 by siRNA induced cell cycle arrest and inhibits cell growth in prostate cancer.^59^ Targeting the PAK6‐LIMK1 axis with miR‐23a suppressed the migration and invasion of prostate cancer cells.^60^ Additionally, inhibition of PAK6 suppressed BAD phosphorylation, which led to apoptosis activation.^61^ Furthermore, the PAK6 protein was found to be highly expressed in hepatocellular carcinoma and to mediate mitosis by regulating Eg5.^62^, ^63^ In cervical cancer, PAK6 has been demonstrated to activate the Wnt/β‐catenin signalling pathway and promote cell growth.^64^ Taken together, these findings indicate that the roles of these hub DEGs and PAK6 as biomarkers or therapeutic targets in GBM cells deserve further exploration.

There were some limitations in the present study. First, we utilized crude extracts obtained from *Vanilla planifolia*, leading us to conclude that VAS, rather than vanillin alone, possesses antitumor effects on GBM cells. Second, we acknowledge that there are some DEGs that warrant further investigation. Despite using RNA‐seq and bioinformatic analysis such as KEGG, fold changes, and STRING, there may still be crucial DEGs in GBM cells that we missed. Third, our objective was to demonstrate the antitumor activity of VAS on GBM cells and identify the involved DEGs. Therefore, further animal models of GBM will be necessary for future in vivo drug development.

## 5 CONCLUSION

In conclusion, we found that the ethanol extract of *Vanilla planifolia* stems significantly reduced the viability and suppressed the colony formation of P#5 TMZ‐R, T98G and U‐87 MG cells. The increase in MAP1LC3 cleavage induced by VAS treatment indicates that it induced autophagy in all three GBM cell lines. According to the RNA‐seq data and bioinformatics analyses, such as KEGG and GO analyses, VAS affected critical functions and pathways related to survival and proliferation in GBM cells. PPI analysis revealed 16 downregulated hub DEGs and 9 upregulated hub DEGs. Further validation of these hub DEGs by qPCR revealed that *DHRS9*, *HOPX*, *AQP5*, *PCP4*, *RGS8*, *GNAT2*, *RLBP1*, *FA2H*, *TNMD*, *SKAP1*, *MATN1*, *IGFBP1*, *ELFN2* and *C2CD4C* were significantly downregulated. Furthermore, the mRNA expression of *IL36RN*, *CCL20*, *CCL5*, *CXCL10*, *HMOX1*, *MX2*, *RSAD2*, *IFI44L* and *EGR1* was significantly upregulated. In addition, the significantly downregulated DEG PAK6 was correlated with the overall survival of GBM patients. Further validation revealed that VAS significantly reduced the mRNA and protein expression of PAK6. Downregulation of PAK6 by the specific shRNAs shPAK#1 and shPAK6#2 led to significant decreases in cell viability and colony formation. Taken together, these findings indicate that VAS has antitumor effects on GBM cells. Moreover, these VAS‐related hub DEGs and PAK6 may be potential therapeutic targets for GBM treatment.

## AUTHOR CONTRIBUTIONS

**Hui Hua Chang:** Funding acquisition (equal); supervision (equal); writing – original draft (equal). **Alice Y. W. Chang:** Supervision (equal). **Bing‐Chen Tsai:** Data curation (supporting). **Yu‐Ju Chen:** Data curation (equal). **Sung‐Ghun Wu:** Data curation (equal). **Li‐Jyun Chen:** Data curation (equal). **Yi‐Xuan Lin:** Data curation (supporting). **Yuan‐Shuo Hsueh:** Conceptualization (lead); funding acquisition (lead); supervision (lead); writing – original draft (lead).

## FUNDING INFORMATION

This work was supported by National Cheng Kung University Hospital (NCKUH‐11202014, NCKUH‐11302031), Kaohsiung Medical University (KMU‐Q113010, 113CM‐KMU‐10, KMU‐TC113A04), the Ministry of Science and Technology (MOST 109‐2314‐B‐309‐002, MOST 110‐2314‐B‐309‐001), and National Science and Technology Council (NSTC 111‐2314‐B‐309‐001, NSTC 111‐2628‐B‐006‐010‐MY3, NSTC 113‐2320‐B‐037‐025) in Executive Yuan, Taiwan.

## CONFLICT OF INTEREST STATEMENT

The authors have no conflict of interest to declare.

## Supporting information


Table S1.

## Data Availability

The original contributions presented in the study are included in the article. Additional files, further inquiries can be directed to the corresponding author.
