# Supplementary material for: Ethanol extract of Vanilla planifolia stems reduces PAK6 expression and induces cell death in glioblastoma cells
Source: J Cell Mol Med. 2024 Sep 4;28(17):e70065. doi: 10.1111/jcmm.70065 (PMC11374694; doi:10.1111/jcmm.70065)
Supplement: Supplementary file 1 — Table S1. [file JCMM-28-e70065-s001.docx]

**Table S1.** Primers used in qPCR analysis.

| Gene name | Sequence | |
| --- | --- | --- |
| PCP4 | Forward | ACATTGACATGGATGCACCAGA |
|  | Reverse | CTGAGACCCAGCCTTCTTCTTC |
| RGS8 | Forward | GAGCCATCCCTGACTTGCTT |
|  | Reverse | CTCCTCTGGCTTTGGGACAG |
| GNAT2 | Forward | AAGAACTGGCCAAGAGGTCC |
|  | Reverse | CCAGCAGTAGCAGCTTGACA |
| RLBP1 | Forward | CCACTTCATCCACCAGCCAT |
|  | Reverse | CCGTGGACAAAGACCCTCTC |
| DHRS9 | Forward | TGGGTGCTAGGCCTCCTAAT |
|  | Reverse | CTGCCAAGTTTCCAAAGCCC |
| PLCB2 | Forward | TGCAGCTCAACTCTGAAGGG |
|  | Reverse | TCCTCCAGGTTTGCCTTTGG |
| HOPX | Forward | CAACAAGGTCGACAAGCACC |
|  | Reverse | GACGGATCTGCACTCTGAGG |
| AQP5 | Forward | CCCGCTCACTGGGTTTTCT |
|  | Reverse | GTCCTCGTCAGGCTCATACG |
| GDA | Forward | GAGCGACAGCGGCAAAATAG |
|  | Reverse | AGCCCAGGCATGAAGAACTC |
| TNMD | Forward | CTGGCATCTACTTCGTGGGT |
|  | Reverse | CTGCTGGGACCCAAATCACT |
| SKAP1 | Forward | ATGCCAGTTACTACCAGGGC |
|  | Reverse | AACAATCCCAACGAGGCTGT |
| MATN1 | Forward | CTGCGAGTCCCTGGTGAAAT |
|  | Reverse | CCGCTTACTCACAGCTTCCA |
| IGFBP1 | Forward | CCTTTGGGACGCCATCAGTA |
|  | Reverse | AGTTCTATTCGGCAGGGCTC |
| ELFN2 | Forward | CCTTTCGCCTCCCTACAAGG |
|  | Reverse | ATGGAACCACTGGACGACAC |
| C2CD4C | Forward | CCCCGTCTTCAACGAGGATT |
|  | Reverse | GCCCTTGTTCACCACCTTGA |
| FA2H | Forward | TACGACCTCTCCAGCTTCGT |
|  | Reverse | TCCCACGTAGTACTGCTCCA |
| IL36RN | Forward | CTCACCTCCAGCTTCGAGTC |
|  | Reverse | ATTCCAGCCACCATTCTCGG |
| CCL20 | Forward | GGCGAATCAGAAGCAAGCAA |
|  | Reverse | GGATTTGCGCACACAGACAA |
| CCL5 | Forward | CGTGCCCACATCAAGGAGTA |
|  | Reverse | TCGGGTGACAAAGACGACTG |
| CXCL10 | Forward | ACTGCCATTCTGATTTGCTGC |
|  | Reverse | ATGCAGGTACAGCGTACAGT |
| HMOX1 | Forward | AGTCTTCGCCCCTGTCTACT |
|  | Reverse | CTTCACATAGCGCTGCATGG |
| MX2 | Forward | ACCGAGCTAGAGCTTCAGGA |
|  | Reverse | TCAGGGGAGGTGATCTCCAG |
| RSAD2 | Forward | TGAGTGTGTTCAGGCAACCT |
|  | Reverse | TTTGGTCTCATCTGGCCCTC |
| IFI44L | Forward | GACTTCTCAAAGCCGGGTCA |
|  | Reverse | CCTTCATGGGGTCCAGTTCC |
| EGR1 | Forward | CCCCGACTACCTGTTTCCAC |
|  | Reverse | TGGGTTTGATGAGCTGGGAC |
| GAPDH | Forward | GTCAAGGCTGAGAACGGGAA |
|  | Reverse | AAATGAGCCCCAGCCTTCTC |
